# Human lung cancer cells express functionally active Toll-like receptor 9

**DOI:** 10.1186/1465-9921-6-1

**Published:** 2005-01-04

**Authors:** Daniel Droemann, Dirk Albrecht, Johannes Gerdes, Artur J Ulmer, Detlev Branscheid, Ekkehard Vollmer, Klaus Dalhoff, Peter Zabel, Torsten Goldmann

**Affiliations:** 1 Medical Clinic, Research Center Borstel, D-23845 Borstel, Germany; 2 Department of Immunology and Cell Biology, Research Center Borstel, D-23845 Borstel, Germany; 3 Department for Thoracic Surgery, Krankenhaus Großhansdorf, D-22927 Großhansdorf, Germany; 4 Clinical and Experimental Pathology, Research Center Borstel, D-23845 Borstel, Germany; 5 Medical Clinic III, University of Lübeck, D-23538 Lübeck, Germany

## Abstract

**Background:**

CpG-oligonucleotides (CpG-ODN), which induce signaling through Toll-like receptor 9 (TLR9), are currently under investigation as adjuvants in therapy against infections and cancer. CpG-ODN function as Th-1 adjuvants and are able to activate dendritic cells. In humans TLR9 has been described to be strongly expressed in B-lymphocytes, monocytes, plasmacytoid dendritic cells and at low levels in human respiratory cells. We determined whether a direct interaction of bacterial DNA with the tumor cells themselves is possible and investigated the expression and function of TLR9 in human malignant solid tumors and cell lines. TLR9 expression by malignant tumor cells, would affect treatment approaches using CpG-ODN on the one hand, and, on the other hand, provide additional novel information about the role of tumor cells in tumor-immunology.

**Methods:**

The expression of TLR9 in HOPE-fixed non-small lung cancer, non-malignant tissue and tumor cell lines was assessed using immunohistochemistry, confocal microscopy, *in situ *hybridization, RT-PCR and DNA-sequencing. Apoptosis and chemokine expression was detected by FACS analysis and the Bio-Plex system.

**Results:**

We found high TLR9 signal intensities in the cytoplasm of tumor cells in the majority of lung cancer specimens as well as in all tested tumor cell lines. In contrast to this non-malignant lung tissues showed only sporadically weak expression. Stimulation of HeLa and A549 cells with CpG-ODN induced secretion of monocyte chemoattractant protein-1 and reduction of spontaneous and tumor necrosis factor-alpha induced apoptosis.

**Conclusions:**

Here we show that TLR9 is expressed in a selection of human lung cancer tissues and various tumor cell lines. The expression of functionally active TLR9 in human malignant tumors might affect treatment approaches using CpG-ODN and shows that malignant cells can be regarded as active players in tumor-immunology.

## Background

The *Toll *gene, the expression of one of it's relatives we are reporting here concerning human malignant tumors, originally was characterized for its role in specifying dorsoventral polarity of the *Drosophila *embryo[1]. Since homologues of *Toll *are also present in plants, mammalian toll-like genes are products of an ancient evolutionary process beginning before the separation of animals and plants [2]. Within the genome of *Drosophila *thus far nine toll-like genes were identified, ten different human toll-like genes are currently described. In contrast to *Drosophila*, the mechanisms taking place in mammalian embryogenesis concerning TLR are widely unknown. The discovery of immune function for *Toll *in *Drosophila *led to a new understanding of innate immunity mechanisms.

Human TLR recognize pathogen-derived products, also termed pathogen-associated molecular patterns (PAMP) [3]. These are bacterial lipoproteins (sBLP) [4], viral double stranded RNA/poly (I:C) [5], lipopolysaccharides (LPS) [6], flagellin [7] and bacterial DNA [8], which engage TLR2, TLR3, TLR4, TLR5 and TLR9, respectively. All functionally characterized TLR signal via the cytoplasmic Toll/interleukin-1 receptor domain (TIR) leading to activation of transcription factors like activator protein-1 (AP-1) and nuclear factor-κB (NF-κB) [9]. TLR9, in contrast to the other TLR, is not located at the cell surface, but intracellularily and, therefore, inhibition of endocytosis or endosome formation completely ablates the effects of CpG-ODN [10].

Different studies show an immunostimulatory capacity of bacterial components which can mediate anti-tumor activity. The first reported use of such a therapy for a nonbacterial disease took place 1890, evaluating the anti-tumor activity of living streptococci directly injected into tumor masses [11]. Shimada demonstrated that bacterial DNA itself can stimulate the immune system [12]. Over the past years there has been an enormous increase in the understanding of the molecular and cellular effects of CpG-ODN [13], which have been found to function as Th-1 adjuvants [14], and are able to activate dendritic cells [15]. This led to the idea to utilize CpG-ODN for induction of anti-tumor immune response as an adjuvant therapeutic strategy [16-18].

In order to characterize possible interactions between malignant cells and CpG-ODN, we investigated whether TLR9 is present in malignant tumors. A variety of malignant solid tumors and cell lines were tested for TLR9 expression; in addition, we examined direct effects of CpG-ODN upon apoptosis and chemokine production of tumor cells.

## Methods

### Tissues

Samples of human tumors and tumor-free tissues were obtained from lobectomies because of lung cancer. Tumor-free tissues were taken at least 5 cm away from the tumor-border. The specimens were fixed and paraffin-embedded using the HOPE-technique [19]. Sections were cut, mounted, and deparaffinized as described elsewhere [20].

For increased comparability of the staining intensities in malignant and non malignant cells we additionally performed IHC on tumor-bearing and tumor free lung tissues which have been assembled on one slide by use of a mechanical tissue arrayer device (MTA1, Alphametrix, Germany).

### Cell culture

A549 cells and HeLa cells were grown in 25 cm^2 ^polystyrene flasks with Dulbecco's modified Eagle's medium DMEM (Sigma) with 10 % heat-inactivated fetal calf serum (PAA Laboratories), 100 μg/ml penicillin G, 100 μg/ml streptomycin and 2 mM L-glutamine (Sigma), maintained under 5 % CO_2 _by routine passage every 3 days. Cells were seeded in 35-mm dishes (Nunc).

For IHC cells were cytocentrifuged and treated by the HOPE-technique [21], the cell lines used were: A549, HeLa, NCI-H727, Jurkat, L428, CPC-N, Raji, H23, U937, H157, H125, L428, and DV90.

### Preparation of the probes

Total RNA was extracted from lung tissues according to the manufacturer's recommendations (RNeasy, Qiagen). After destroying residual DNA with DNase (Invitrogen), cDNA was synthesized by reverse transcription [22]. PCR was performed targeting a 393 bp fragment of human TLR9-mRNA (TLR9 forward: AAC TGG CTG TTC CTG AAG TC; TLR9 reverse: TGC CGT CCA TGA ATA GGA AG). PCR-products were separated on 2 % agarose gels stained by ethidiumbromide. Cycle sequencing confirmed 100 % identity with the human TLR9 wild-type-sequence. Probes were labeled with digoxigenin using High-Prime (Roche) according to the manufacturer's recommendations [23].

### ISH

Hybridization, detection of signals and controls were carried out as previously described (concentration of probe 2 ng/μl, hybridization temperature 46°C) [20,22].

### IHC

Primary antibody (mouse anti-human TLR9, clone 26C593, Imgenex) was applied in a dilution of 1/100 in PBS for 16 h at 4°C. Negative controls comprised omission of the primary antibody. Detection was performed by horseradish-peroxidase labeled streptavidine-biotin technique (LSAB2, Dako) [24].

### RT-PCR/Cell lines

**Figure 1:**
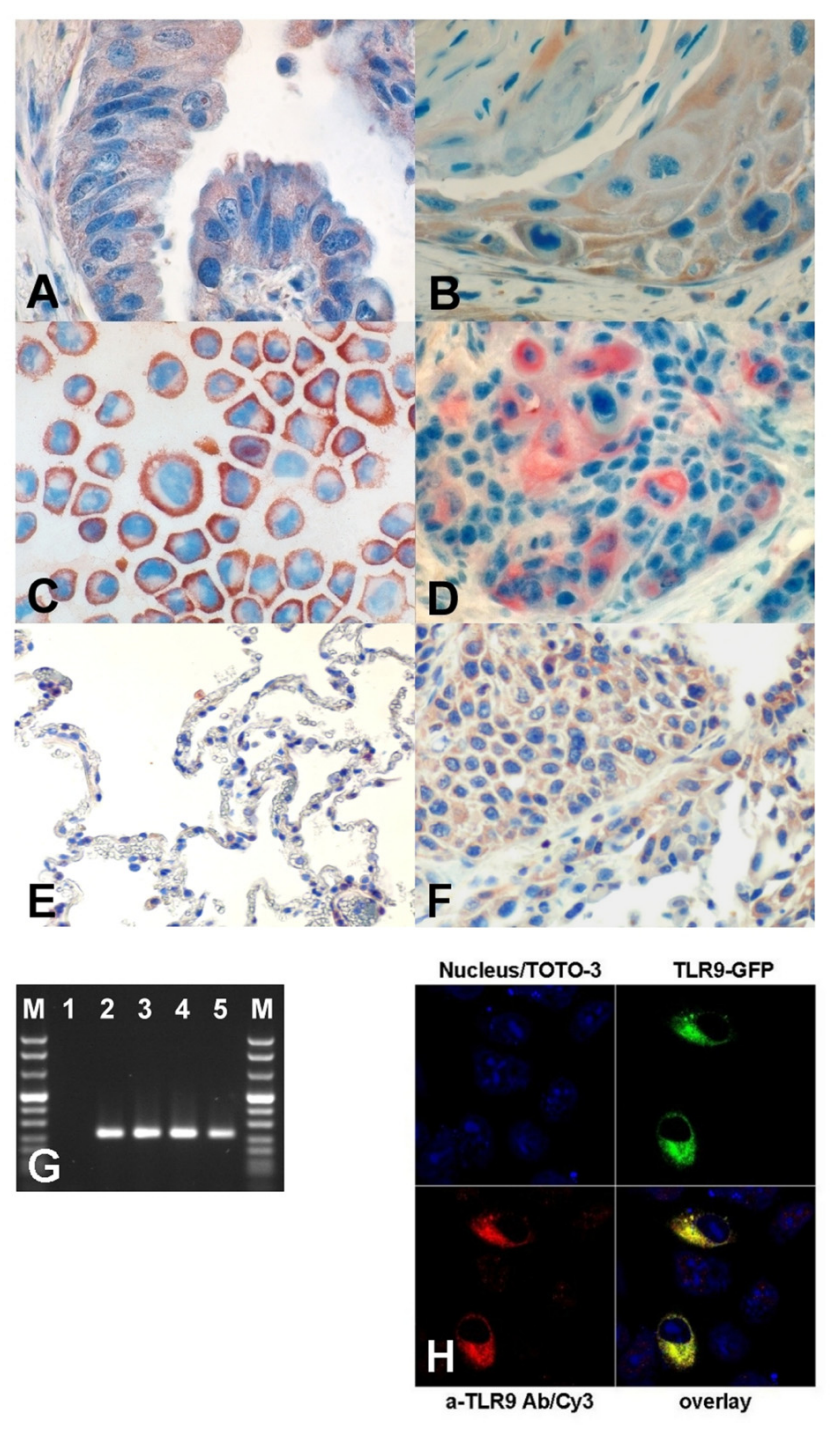
Immunohistochemistry (IHC) (A-C) for TLR9 detected by a mouse monoclonal antibody. Adenocarcinoma of the lung **(A)**. Squamous cell carcinoma of the lung **(B)**. A549 cells (all 600 ×) **(C)**. *In situ *hybridization (ISH) targeting mRNA of human TLR9 with a digoxigenin-labeled DNA-probe in a squamous cell carcinoma of the lung (600 ×) **(D)**. Immunohistochemical staining of TLR9-expression-levels in nonmalignant **(E) **and malignant tissues **(F) **derived from the same lungs an stained by the use of tissue arrays. Results of RT-PCR targeting TLR9 in cell lines **(G)**. M: molecular-weight marker (MW8, Roche). 1: negative control; 2: A549; 3: NCI-H727; 4: BEAS 2b; 5: Mononuclear cells from a healthy human donor. Confocal laser microscopy of A549 cells transiently transfected with a GFP-TLR9 plasmid: Cytoplasmic expression of TLR9 is observable in all cells, while successful transfection led to overexpression of TLR9 resulting in bright GFP signals completely superimposed by the TLR9 antibody signal **(H)**. Nuclear counterstain was performed with TOTO3.

A549, HeLa, BEAS 2b, U937, and NCI-H727 cell lines were used. RT-PCR was performed like described above using TLR9 specific primers (forward: 5'CATGCCCTGCGCTTCCTATTCA; reverse: 5'TGGGCCAGCACAAACAGCGTCTT) spanning an amplicon of 260 bp. Mononuclear cells were included as positive control as well as RT-PCR targeting glyceraldehyde-3-phosphate dehydrogenase (GAPDH) (forward: GTCATCATCTCCGCCCCTTCTGC; reverse: GATGCCTGCTTCACCACCTTCTTG) (not shown). PCR-products were separated along with a molecular weight marker (MW8, Roche) using 2 % agarose gels (Fig. 1).

### Transfection

A549-cells were seeded in 35-mm glass bottom dishes (MatTek Corp.) overnight. Cells were transfected with GFP-huTLR9 using Polyfect (Qiagen) according to the manufacturer's instructions or incubated in medium.

### Confocal Microscopy

Cells were washed in tris-buffered-saline, containing 0.2 % Tween 20 (TTBS), fixed with 4 % paraformaldehyde in phosphate-buffered-saline (PBS) for 10 min on ice, and permeabilized with 0.25 % Triton-X100 (Roche) in PBS for 10 min. Cells were washed with TTBS, blocked with 10 % bovine-serum-albumine (BSA) in TBS for 20 min, and incubated with primary antibody (clone 26C593, Imgenex) or isotype (Mouse IgG1, Jackson ImmunoResearch Laboratories) 1:150 in TBS 10 % BSA for 30 min. Cells were washed with TTBS, incubated for 30 min with Alexa-568/goat-anti-MouseIgG1 (Molecular Probes Inc.) 1:500 in TBS containing 10 % BSA, and washed with TTBS. Counterstaining was achieved using TOTO-3 1:500 in TBS containing 10 % BSA. Cells were washed with TTBS, fixed again as above, mounted and analyzed using a confocal laser microscope. The GFP-TLR9 plasmid was kindly provided by Terje Espevik, Trondheim, Norway.

### Treatment Protocols

For CpG-ODN stimulation the M362 sequence was used in a concentration of 1 μM; as control M383 was used as described by Hartmann et al. [25] (MWG-Biotech). Human tumor necrosis factor-alpha (TNF-α, Roche) in PBS containing 0.5 % bovine serum albumin was added to the cultures in a concentration of 10 ng/ml. CHX (Sigma) was dissolved in PBS and added in a concentration of 10 μM.

### Flow cytometry

Annexin-V FITC apoptosis kit I and PE-conjugated active caspase-3 apoptosis kit I were used according to the manufacturer's instructions (BD Pharmingen). TLR9 antibody and isotype control (eBioscience, clone: eB72-1665) were stained after fixation and permeabilization using Intraprep (Beckmann Coulter) according to the manufacturer's instructions. Flowcytometric data (FACS Calibur) collected from 10,000 cells are reported as percentages of positive cells (Becton Dickinson).

### Cytokine assays

Cell culture supernatant (50 μl per sample) was analyzed using the Bio-Plex system and a Luminex 100TM analyzer (BioRad) according to manufacturer's instructions.

### Stimulation of tumor-tissues and RT-PCR

**Figure 4:**
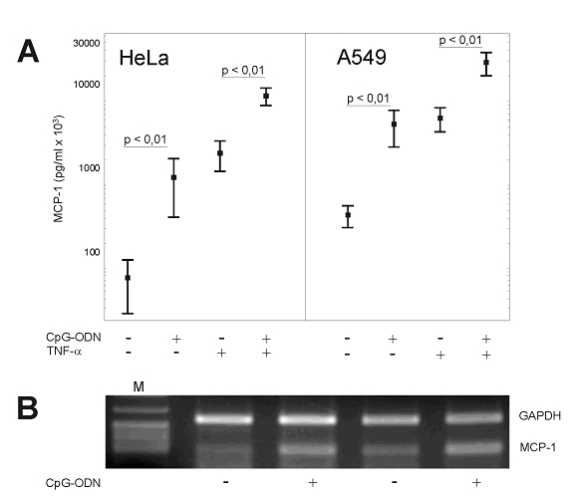
MCP-1 secretion in response to CpG-ODN-stimulation in the presence or absence of TNF-α by HeLa and A549 cells **(A)**. Data are expressed as the mean ± SD (n = 6). Student's *t *test was used for statistical analysis. RT-PCR targeting mRNA of MCP-1 in human non-small cell lung cancer tissue stimulated with CpG-ODN for 24 h **(B) **(M = pBR322-*Msp1*). Lanes 2 and 3, as well as lanes 4 and 5 respectively show results of tissue samples from the same tumors either in the absence or presence of CpG-ODN.

Tissue blocks from lung cancer specimens (edge length approximately 0.5 cm) were cultivated in RPMI 1640 at 37°C and 5 % CO_2 _for 24 h, and either stimulated or not stimulated with 1 μM of CpG-ODN (M362 sequence). These blocks from adjacent locations of the same lung-tumors were fixed using the HOPE-technique and paraffin embedded. RT-PCR was carried out like described above using primers targeting human MCP-1 (forward: AAAGCACCAGTCAACTGGAC; reverse: AGCGCTTGGTGATGTGCTTT) resulting in a 149 bp PCR-product and GAPDH (forward: AGAACGGGAAGCTTGTCATC; reverse: TGCTGATGATCTTGAGGCTG) resulting in a 257 bp PCR-product. PCR products were separated on 2 % agarose gels along with a molecular weight marker (pBR322-*Msp1*) and the results displayed in figure 4b.

## Results

### Expression of TLR9 in malignant tumors

To investigate the expression of TLR9 in human lung tumors and lung tumor cell lines we used the recently described HOPE-fixation method. HOPE-fixed [19] specimens showed superior preservation of morphology after *in situ *hybridization (ISH). The generation of TLR9-signals was achieved within 10 minutes, whereas unspecific signals were not detected in the control preparations. We found high signal intensities for TLR9 transcripts in the cytoplasm of tumor cells in the majority of lung cancer specimens. Immunohistochemistry (IHC) revealed strong TLR9 protein expression within tumor cells of tissues and cell lines. In contrast normal lung tissues sporadically showed weak expression of TLR9 mainly in cells revealing morphological characteristics of alveolar macrophages and alveolar epithelial cells as displayed in figure 1. Negative control specimens did not display signals. The results are summarized in table 1; some representative results of ISH and IHC are displayed in figure 1. To confirm the results obtained by ISH we analyzed TLR9-transcripts in tumor cell lines by RT-PCR. As shown in figure 1, we found that all tumor cell lines indeed express TLR9.

**Table 1:** Summarized results of immunohistochemistry (IHC) targeting TLR9 in tumor tissues and cell lines.

**Entity**	N*	**No expression**	**Weak expression**	**Strong expression**
**Adenocarcinoma of the lung**	21	1	7	13
**Squamous cell carcinoma of the lung**	23	1	14	8
**Large cell carcinoma of the lung**	3	0	2	1
Cell lines**	13	0	1	12
**Total**	60	2	24	34

* Number of analyzed specimens

** See methods

A cytoplasmic localization of TLR9 was confirmed by confocal microscopy (fig. 1). This finding is in agreement with previous studies on the distribution of TLR9 in RAW264.7 cells [10]. Furthermore, immunostaining of GFP-TLR9 transfected A549 cells verified the specificity of the TLR9 antibody: Only those cells which were successfully transfected as demonstrated by the GFP-dependent fluorescence also stained brightly with the TLR9 antibody.

### CpG-ODN stimulation reduces spontaneous and tumor necrosis factor-alpha (TNF-α)/Cycloheximide (CHX)-induced apoptosis

**Figure 2:**
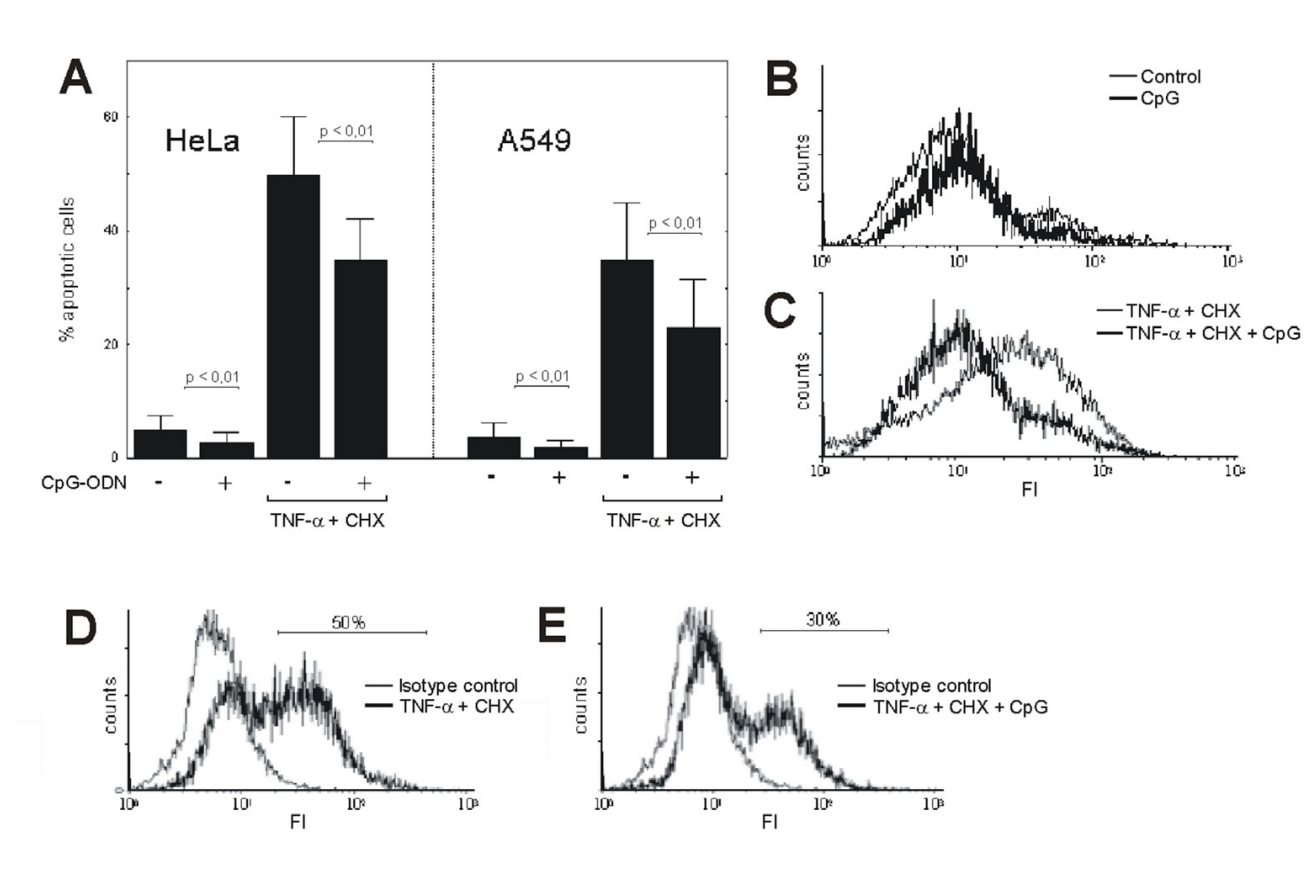
CpG-ODN-stimulation decreases apoptosis in HeLa and A549 cells. Cells were stained with Annexin-V after CpG-ODN-stimulation in the presence or absence of TNF-α and CHX after 24 h **(A)**. Data are expressed as the mean ± SD (n = 6). Student's *t *test was used for statistical analysis. Representative histograms are shown from experiments with HeLa cells after CpG-ODN-stimulation in the absence **(B) **or presence **(C) **of TNF-α and CHX. Caspase 3 expression in HeLa cells is shown after incubation with TNF-α and CHX **(D)**. In the presence of CpG-ODN the expression is decreased **(E)**. The percentage of positive cells in each sample is indicated.

The expression of TLR9 in tumor cells and cell lines rises up the question, whether this receptor is functional active in these cells. As shown in figure 2a, CpG-ODN decrease the rate of spontaneous and induced apoptosis in HeLa and A549 cells after treatment with TNF-α and CHX. Representative histograms demonstrate the detection of annexin in the presence or absence of CpG-ODN and TNF-α/CHX (Fig. 2b and 2c). The induction of apoptosis after stimulation with TNF-α/CHX was further verified by the expression of active caspase 3 as shown in figure 2d. In the presence of CpG-ODN the expression was reduced analogous to the reduction of annexin-staining (Fig. 2e).

### Influence of induced apoptosis on TLR9 expression

**Figure 3:**
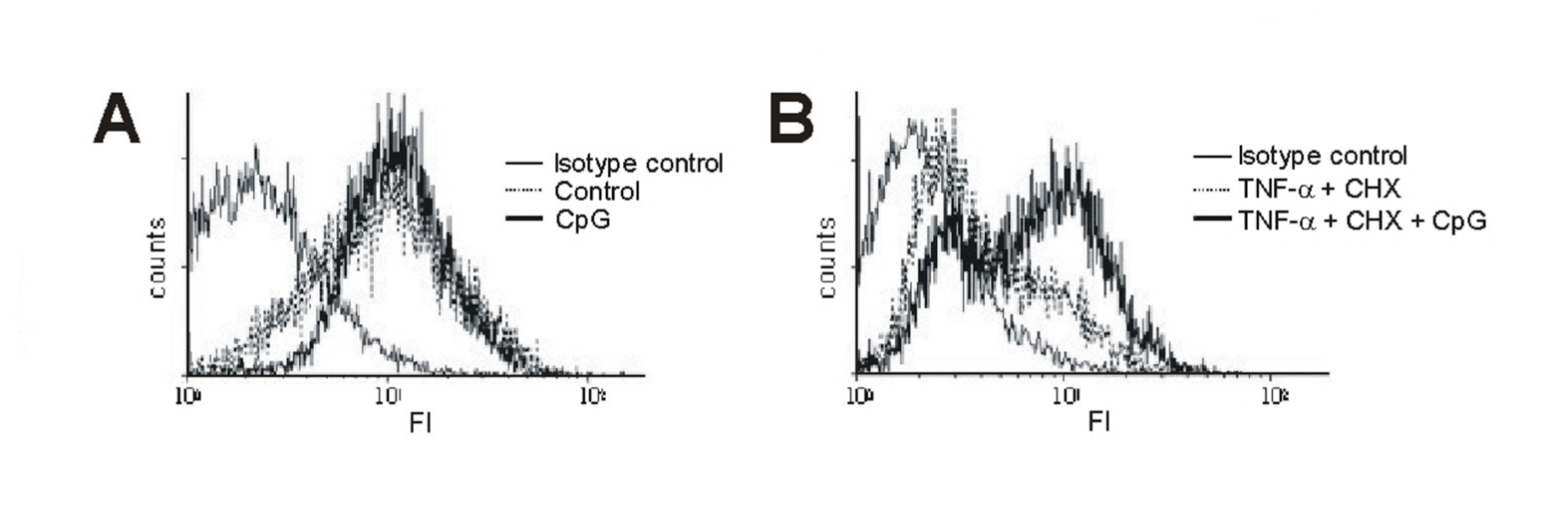
TLR9 expression after CpG-ODN-stimulation in HeLa cells: There is no difference in TLR9 expression with and without CpG-ODN-stimulation after 24 h **(A)**. CpG-ODN partially inhibit downregulation of TLR9 which is induced by TNF-α and CHX **(B)**. FI = fluorescence intensity.

Here we investigated, whether CpG-ODN can modulate their own receptor. We found no differences in TLR9 expression with and without CpG-ODN stimulation. However, in the presence of TNF-α/CHX the expression of TLR9 was strongly reduced, whereas CpG-ODN stimulation counteracted this downregulation (Fig. 3a and 3b).

### Secretion of MCP-1 in response to CpG-ODN and TNF-α

In order to obtain further information about the functional activity of TLR9 in tumors we studied cytokine release upon CpG-ODN stimulation. The measurement of cytokines from stimulated HeLa and A549 cells revealed a significantly enhanced release of monocyte chemoattractant protein-1 (MCP-1) after 24 h of stimulation in response to CpG-ODN or TNF-α (Fig. 4a). The production was further enhanced when stimulated with a combination of CpG-ODN and TNF-α (Fig. 4a). There was no effect of CpG-ODN on TNF-α production (data not shown). To verify the induction of MCP-1 by CpG-ODN in cell lines we additionally analyzed human tumor tissues by RT-PCR; the results are shown in figure 4b. The relative amounts of RT-PCR-signals for MCP-1 in relation to GAPDH were higher in the specimens treated with CpG-ODN if compared with the controls confirming the results obtained in cell culture experiments on the tissue level.

## Discussion

By application of a novel fixation technique we specify for the first time the expression of TLR9 protein and mRNA in a selection of human non small cell lung cancer tissues as well as cell lines. Stimulation of the TLR-9 expressing cell lines A549 and HeLa with CpG-ODN showed a marked antiapoptotic effect. In addition, there was substantially enhanced release of MCP-1 from the cell lines upon CpG-ODN stimulation which was also shown in *ex vivo *experiments. We conclude the expression of a functionally active TLR9 in human malignant tumors.

The presence of molecules involved in ontogenesis e.g. the carcinoembryonic antigen (CEA) is frequently observed in malignant tumors suggesting a kind of "shift-back" towards earlier developmental stages [26]. The significance and underlying mechanisms of this phenomenon are poorly understood; nevertheless, the detection of such molecules is used for diagnostic purposes in cancer [27]. The role of TLR in mammalian embryogenesis is unknown, and thus far there is no evidence for an endogenous TLR9 ligand homologous to *Spaetzle*. Such a ligand could play a role for the activation of human TLR9. Whether the expression of TLR9 in human malignant cells takes advantage of TLR9-function in embryogenesis therefore remains unclear.

On the other hand TLR9 in malignant cells could have similar functions as in cells of the innate and adaptive immune system. In humans TLR9 has been described to be mainly expressed in B-lymphocytes, monocytes and plasmacytoid dendritic cells [28]. In addition Platz et al. reported a weak expression in respiratory epithelial cell lines and primary epithelial cells [29].

The CpG-ODN sequence M362 used in our study is known to potently activate TLR9-expressing immune cells in humans including plasmacytoid dendritic cells and B cells as shown by Hartmann et al. [25] B cells are induced to proliferate and secrete immunoglobulin in response to CpG-ODN, dendritic cells produce a wide array of cytokines and apoptosis is inhibited [30,31].

These mechanisms are both reflected in the results we obtained in our study after CpG-ODN stimulation of malignant cells:

Firstly, stimulation of the A549 and HeLa cells with CpG-ODN showed an antiapoptotic effect. This was demonstrated for spontaneous as well as induced apoptosis with TNF-α and CHX after 24 h. Our observation is consistent with previous evidence in other cell lines. Yi et al. demonstrated antiapoptotic effects of CpG-ODN in a mouse B lymphoma cell line [32], and similar changes were described in chronic lymphocytic leukemia cells [33,34]. Previous data of systemic administration of bacterial DNA as a single agent *in vivo *showed anti-tumor effects. However, this anti-tumor effect appears to be effective indirectly and is related to enhanced NK cell activity. In a murine model of lymphoma the immunostimulatory effect of CpG-ODN was demonstrated to be responsible for the observed anti-tumor effects [35]. Carpentier *et al. *have shown that CpG-ODN *in vivo *induced rejection of neuroblastoma xenografts [36]. In contrast CpG-ODN had no effect on survival in mice inoculated with the 38C13 murine B cell lymphoma. However, a single injection of CpG-ODN enhanced the response to anti-tumor antibody therapy [37]. To what extent the antiapoptotic effects of CpG-ODN on tumor cells demonstrated in our study affect the tumorbiology *in vivo *requires further investigation.

Secondly, tumor cell lines (A549 and HeLa) stimulated with CpG-ODN showed strong secretion of the CC chemokine MCP-1. Furthermore a similar effect was observed in the investigated tumor tissues. Immunostimulatory properties together with anti-tumor activity of bacterial DNA were initially reported for a DNA fraction derived from mycobacteria by Tokunaga and coworkers [38]. It is known that such DNA induces enhanced production of various cytokines with anti-tumoral activity in NK cells, B cells, monocytes, macrophages and dendritic cells, such as TNF-α, IL-12, and IFN-γ [39]. In our study a substantial costimulatory effect in addition to CpG-ODN was achieved using TNF-α. MCP-1 has various biological activities including the induction of increased cytotoxic activity of monocytes and NK cells. Transfection of MCP-1 into a human malignant glioma cell line tested on nude mice did not reduce the tumor mass but was associated with the infiltration of large numbers of NK cells and monocytes at the tumor site [40]. A further study by Nokihara et al. performed with transfection of the MCP-1 gene into human lung adenocarcinoma cells showed reduced systemic spread of transfected cells inoculated i.v. in NK cell-intact severe combined immunodeficient (SCID) mice. These findings suggest that locally produced MCP-1 suppresses tumor progression by a NK cell-mediated mechanism [41]. Thus, apart from the direct activation of immune cells, the effect of CpG-ODN stimulation on the secretion of MCP1 by TLR9 expressing tumor cells could possibly lead to anti-tumoral effects due to an increase of local MCP1 production which then might lead to attraction of immune cells. The costimulatory effect of TNF-α as demonstrated *in vitro *in this study could further enhance this scenario.

Regarding TLR9 expression in nonmalignant lung tissue our data confirm the findings of low TLR9 expression in respiratory cells of Platz et al. [29], who have been working on single cell preparations. However TLR9 expression was only seen sporadically weak in nonmalignant lung tissue.

Biological explanations for the TLR9 expression in malignant cells require further investigations. Three possibilities are conceivable: Either this could represent a bystander phenomenon, a side effect of a pathway functional to a different purpose. Secondly the upregulation of TLR9 could be beneficial to the tumor, promoting tumor cell survival. Thirdly, it even might help immune control strategies of the organisms an element of a pathway directing defense mechanisms against malignantly transforming cells. While the first possibility seems unlikely in the light of our findings of a functionality of the receptor in various *in vitro *and *ex vivo *experiments, our data provide evidence for the second as well as the third possibility; the sum effect of these two counteracting mechanisms in an *in vivo *setting can not be estimated from these experiments and could even differ from tumor entity to tumor entity.

## Conclusions

In conclusion, we showed in a selection of samples that human malignant tumors express functionally active TLR9 and respond to CpG treatment with prolonged survival and chemokine release. This might influence the effects of CpG-ODN based anti-tumor therapies. Broad screening approaches will be worthwhile to further substantiate these initial results.

While recent strategies in tumor-immunology mainly target a strengthening of the host-defense, we provide evidence that the malignant cells themselves can be regarded active players in the complex struggle between tumor and host. In any case CpG-ODN based anti-tumor therapies should be reconsidered in the light of our findings since CpG-ODN products are currently in Phase I/II clinical trials both as a monotherapy and as part of multi-drug regimens.

## Author's contributions

DD carried out the flow cytometry and cytokine assays and was involved in the design and coordination of the study and drafting the manuscript. DA and AJU carried out the confocal microscopy, RT-PCR with cell lines and were involved in drafting the manuscript. JG was involved in immunohistochemistry of cell lines and the design of the study. DB conducted the surgical part of the study. EV conducted the pathological part of the study and was involved in the design of the study. KD and PZ conducted the clinical part of the study and were involved in the design and coordination of the study. TG performed the immunohistochemistry, in situ hybridization and RT-PCR with tissues and conceived of the study. All authors read and approved the final manuscript.
